# Expression of insulin-like growth factor I and its receptor in the liver of children with biopsy-proven NAFLD

**DOI:** 10.1371/journal.pone.0201566

**Published:** 2018-07-31

**Authors:** Anna Alisi, Valentina Pampanini, Cristiano De Stefanis, Nadia Panera, Annalisa Deodati, Valerio Nobili, Stefano Cianfarani

**Affiliations:** 1 Research Unit of Multifactorial Genetics and Epigenetics, “Bambino Gesù” Children’s Hospital–IRCCS, Rome, Italy; 2 NORDFERTIL Research Lab Stockholm, Paediatric Endocrinology Unit, Department of Women’s and Children’s Health, Karolinska Institutet and University Hospital, Solna, Sweden; 3 Histology-Core Facility “Bambino Gesù” Children’s Hospital–IRCCS, Rome, Italy; 4 Dipartimento Pediatrico Universitario Ospedaliero “Bambino Gesù” Children’s Hospital–IRCCS, Tor Vergata University, Rome, Italy; 5 Department of Pediatric- University “La Sapienza”, Rome, Italy; 6 Hepato-Gastroenterology Disease Unit, “Bambino Gesù” Children’s Hospital–IRCCS, Rome, Italy; University of Navarra School of Medicine and Center for Applied Medical Research (CIMA), SPAIN

## Abstract

**Background and aims:**

Nonalcoholic fatty liver disease is one of the major complications of obesity, occurring already in pediatric age. Insulin like growth factor-I has been proposed as a potential therapeutic agent for its beneficial effect in experimental liver fibrosis. The aim of this work was to investigate the expression of insulin-like growth factor-I and its receptor in the liver of children with biopsy-proven nonalcoholic fatty liver disease and relate it to liver histological features.

**Methods:**

45 obese children and adolescents (14 females and 31 males) with nonalcoholic fatty liver disease were included. Insulin like growth factor-I and its receptor expression was evaluated in liver tissue by immunofluorescence and qPCR.

**Results:**

The expression of insulin like growth factor-I and its receptor were significantly related to fibrosis and were higher in children with stage 3 fibrosis compared to stage 1 and 2 (p<0.001 and p = 0.007 respectively). mRNA of insulin like growth factor-I receptor was higher in more advanced stages of fibrosis (p<0.001). Furthermore, the expression of insulin like growth factor-I and its receptor in hepatic stellate cells, the cell type mostly involved in fibrosis progression, was significantly increased in stage 3 fibrosis compared to stage 1 (p = 0.01 and p = 0.008 respectively).

**Conclusions:**

We demonstrated for the first time that insulin like growth factor-I and its receptor are upregulated in children with nonalcoholic fatty liver disease. These findings give a new hint for the potential therapeutic use of insulin like growth factor-I in pediatric nonalcoholic fatty liver disease complicated by liver fibrosis.

## Introduction

Nonalcoholic fatty liver disease (NAFLD) is one of the major co-morbidities associated with the obesity [1]. The term encompasses a spectrum of hepatic conditions ranging from simple steatosis to nonalcoholic steatohepatitis (NASH), fibrosis and possible progression into cirrhosis and hepatocellular carcinoma [2]. Prevalence of NAFLD has greatly increased during the last decades in both children and adults with an obese phenotype [3, 4].

The pathogenesis of NAFLD is characterized by two main phases [5]: the intra-hepatic lipid accumulation that could be driven by insulin resistance and lipotoxicity [6]; the development of NASH and fibrogenesis that depends on several mechanisms including adipocytokine imbalance, oxidative stress and gut dysbiosis-mediated endotoxemia [7, 8].

The activation of hepatic stellate cells (HSCs) plays a key role in fibrogenesis, the process that lead to fibrosis. HSCs are pericytes-like cells situated in contact with both endothelial cells and hepatocytes in the perisinusoidal space. Upon stimulation by inflammatory molecules, these cells activate into myofibroblasts that express α-smooth muscle actin (α-SMA) as hallmark [9]. Activated HSCs acquire a pro-inflammatory and fibrogenic phenotype and are able to migrate to the sites of liver injury where they produce large amounts of extracellular matrix molecules, such as collagen, and induce liver fibrosis [9]. In pediatric populations, where up to approximately 80% of obese individuals are affected by NAFLD, fibrosis is a histologic trait of disease, even if the advanced stage of fibrosis and cirrhosis rarely occur [10, 11].

There are no pharmacological treatments currently licensed for NAFLD and the weight loss *via* lifestyle interventions remains the mainstay of treatment [12]. However, several studies in children with NAFLD demonstrated that even if lifestyle interventions are able to revert steatosis [13–15], they are often ineffective on liver tissue damage, particularly on fibrosis that is currently the major target for designing novel therapies for NASH.

A second major issue in NAFLD is the diagnosis of NASH and fibrosis. To date the gold standard for the diagnosis and staging of NAFLD is liver biopsy that can expose the child to a series of risks [16]. Therefore, the identification of novel potential non-invasive biomarkers of NASH and fibrosis is challenging.

In the last two decades, growth hormone (GH)/insulin-like growth factor-I (IGF-I) axis has been investigated for its putative role in liver diseases. NAFLD prevalence is higher in patients with GH deficiency (GHD) [17, 18] and GH treatment has proven effective in reducing liver fibrosis [18, 19] and reversing NASH [20] in these patients. *In vivo* models of experimental cirrhosis have contributed to the knowledge from clinical studies, showing that IGF-I acts as anti-inflammatory, anti-oxidant, anti-fibrogenic and hepatoprotective molecule [21–25]. IGF-I does not affect hepatocyte function directly because the hepatocytes express few IGF-I receptor (IGF-IR) in normal condition [26]. On the other hand, the upregulation of IGF-IR in liver during pathologic conditions has been widely reported [27–29]. Moreover, IGF-IR is strongly expressed in activated HSCs and IGF-I-induced cellular senescence in HSCs, *in vitro* and *in vivo* [26, 30].

Cross-sectional studies in adult patients have demonstrated an association between circulating levels of GH and IGF-I and NAFLD severity [31–33], leading to speculative hypotheses about a role of these molecules as predictive markers or therapeutic targets in NAFLD. However, studies in pediatric cohorts are limited. We have previously demonstrated a relationship between circulating IGF-I and liver histological stages in a large cohort of obese children with NAFLD, suggesting that IGFs may be markers of liver damage progression in obese children [34].

Little is known about the IGF system status in the liver tissue of obese patients with NAFLD. The aim of the present study was to investigate IGF-I and IGF-I receptor (IGF-IR) expression in the liver of children with biopsy-proven NAFLD and relate it to liver histological features.

## Materials and methods

### Study population and design

A cohort of 45 Caucasian obese children and adolescents (14 females, 31 males, mean age 12.2 ± 3.0 y) with diagnosis of NAFLD who were referred to the Hepatometabolic Unit of Bambino Gesù Children’s Hospital between January 2012 and July 2014 was included in the study. The study was conducted in accordance to the guidelines of the 1975 Declaration of Helsinki. Written informed consent was obtained from all children and their parents. The experimental protocol and the process for obtaining informed consent were approved by the Institutional Review Board of Bambino Gesù Children's Hospital.

Eligibility criteria included obesity, established using body mass index (BMI) cutoffs of the International Obesity Task Force; absence of underlying diseases and medications that could influence the outcome measures (i.e. GH therapy); all 4 grandparents of Italian descent. Maternal diabetes (either preexisting or developed during or after the index pregnancy) was the sole exclusion criterion. After obtaining informed consent, we performed in-person study visits. At the in-person visit, trained research assistants measured height to the nearest 0.1 cm using a calibrated stadiometer and weight to the nearest 0.1 kg using a calibrated scale. Each child’s BMI was calculated using the following formula: BMI = weight (in kg)/height (in m^2^). The anthropometric data were compared with national standards. Waist circumference (WC) was measured using a tape measure at just above the uppermost lateral border of the right ilium, at the end of a normal expiration, as described by the National Center of Health Statistics.

### Diagnosis of NAFLD and liver histopathology

All children underwent liver biopsy for persistently elevated serum aminotransferase level and/or diffusely hyperechogenic liver on ultrasonography and after exclusion of other common or less common liver diseases, including hepatitis B virus, hepatitis C virus and autoimmune hepatitis, celiac disease, genetic disorders (such as Wilson's disease, alpha-1-antitrypsin deficiency) hemochromatosis, hepatic malignancies and drug-induced liver disease [35].

The clinical indication for biopsy was to assess the presence of NASH and fibrosis or other likely independent or competing liver diseases. Liver biopsy was performed in all children, after an overnight fast, using an automatic core biopsy 18-gauge needle (BioPince; Amedic, Kista, Sweden) under general anesthesia and ultrasound guidance. The length of the liver specimen (in millimeters) was recorded. Only samples that were not fragmented, at least 15 mm long, and included at least 6 complete portal tracts were considered adequate for use in this study. Biopsy specimens were processed routinely (ie, formalin- fixed and paraffin-embedded), and 5-mm-thick liver tissue sections were stained with hematoxylin and eosin, van Gieson, periodic acid-Schiff diastase, and Prussian blue stains. Liver biopsy specimens were evaluated by a single expert pediatric hepatopathologist who established the histopathological diagnosis of NASH. To determine the intraobserver agreement, the pathologist scored the biopsy specimens twice while blinded and calculated the weighted k coefficients for various histological features. Steatosis, inflammation, hepatocyte ballooning, and fibrosis were scored based on the NAFLD Clinical Research Network criteria [36]. Steatosis was graded on a 4-point scale: grade 0, steatosis involving 66% of hepatocytes. Lobular inflammation also was graded on a 4-point scale: grade 0, no foci; grade 1, 4 foci per 200 x field. Hepatocyte ballooning was graded from 0 to 2: 0, none; 1, few balloon cells; 2, many/prominent balloon cells. The stage of fibrosis was quantified on a 5-point scale: stage 0, no fibrosis; stage 1, perisinusoidal or periportal (1a, mild, zone 3, perisinusoidal; 1b, moderate, zone 3, perisinusoidal; 1c, portal/periportal); stage 2, perisinusoidal and portal/periportal; stage 3, bridging fibrosis; stage 4, cirrhosis. Features of steatosis, lobular inflammation, and hepatocyte ballooning were combined to obtain the NAFLD activity score (NAS). As recommended by the NASH Clinical Research Network, a microscopic diagnosis based on overall injury pattern (eg, steatosis, hepatocyte ballooning, inflammation) as well as the presence of additional lesions (eg, zonality of lesions, portal inflammation, fibrosis) was assigned to each case.

### RNA extraction and real-time polymerase chain reaction (RT-PCR)

In formalin-fixed paraffin-embedded (FFPE) tissue collected from 18 out of 45 NAFLD patients we evaluated the mRNA expression levels of *IGF-1R* by RT-PCR. Purification of total RNA was performed using RNeasy FFPE kit, (Qiagen Valencia, CA 91355, USA) combining for each sample 4 section up to 10 μm. FFPE tissue sections were deparaffinized by xylene and ethanol washes. After evaporation of residual ethanol, sections were incubated at 56ºC for 15 min and then 80°C for 15 min with proteinase K buffer and proteinase K for sample lysis and partial reverses formalin crosslinking. The supernatants were collected in a new micro-centrifuge tube and incubated with DNase Booster buffer and DNase at room temperature for 15 minutes. The last steps of RNA purification were performed using MinElute spin columns according to the manufacturer’s specification.

Total RNA (500 ng for each sample) was converted to cDNA using High-Capacity cDNA Reverse Transcription Kit (Thermo Fisher Scientific—Applied Biosystems, Carlsbad, CA, USA) and used as starting material for gene expression study by real-time PCR assay.

SensiFAST Probe Hi-ROX One-Step kit (Bioline USA Inc., MA, USA), Applied Biosystems Taqman Gene Expression Assay for *IGF1R* (ID: Hs00609566_m1) and 7500 Fast Real-Time PCR System were employed for gene expression analysis. The house keeping gene *18S* (ID: Hs99999901_s1) (Thermo Fisher Scientific—Applied Biosystems, Carlsbad, CA, USA) was used as an endogenous control.

For each sample, the measures were done in triplicate for both *IGF1R* and *18S*. Finally, the mRNA levels were relatively quantified using the ΔΔCT method considering patients with fibrosis of grade as value = 1.

### Immunohistochemistry and immunofluorescence

Immunofluorescence was performed on 2 μm-thick sections obtained from formalin-fixed tissue embedded in paraffin. Antigen retrieval was performed with ethylenediaminetetraacetic acid (EDTA) (pH 9) (Dako, Glostrup, Denmark).

Sections were incubated over night at +4°C with anti-human: rabbit polyclonal anti-IGF-I antibody (dilution 1:300; Abcam, Cambridge, UK); rabbit polyclonal anti-IGF-IR antibody (dilution 1:50; Abcam, Cambridge, UK); mouse monoclonal anti-α-SMA antibody (dilution 1:100 Dako, Glostrup, Denmark).

The slides where then revealed with mouse anti-rabbit Alexa Fluor 488 (1:500, Applied Biosystems, Life Technologies, Carlsbad, CA, USA) and goat- anti-mouse Alexa Fluor 555 (1:500, Applied Biosystems, Life Technologies, Carlsbad, CA, USA).

Nuclei were counterstained with 4',6-Diamidino-2-phenylindole dihydrochloride (DAPI) for 5 minutes after extensive washing. Next sections were mounted with PBS/glycerol (1:1) and covered with a coverslip.

The confocal microscopy imaging was performed on Olympus Fluoview FV1000 confocal microscope equipped with FV10-ASW version 2.0 software, using 20× objective. Optical single sections were acquired with a scanning mode format of 1024 × 1024 pixels, sampling speed of 40 μs/pixel, and 12 bits/pixel images. Fluorochromes unmixing was performed by acquisition of automated-sequential collection of multi-channel images, in order to reduce spectral crosstalk between channels.

For quantification of expression were used at least five free area from single section that was used to manually draw the region of interest (ROI). The intensity average of fluorescence was calculated using NIS-Elements BR Analysis.

### Statistical analysis

Results are expressed as mean ± SD unless specified otherwise. Correlations were calculated as Spearman correlation coefficients. Fisher Exact test was used to calculate differences between proportions. The continuous variables among groups were compared by ANOVA on ranks test. A P value < 0.05 was considered statistically significant. All analyses were performed using the SigmaStat (v 11.00) package (SPSS, Inc, Chicago, IL, USA).

## Results

### The expression IGF-I and IGF-IR in liver tissue correlates with fibrosis

The main demographic, anthropometric and biochemical features of the study population are reported in Table 1.

**Table 1:** Anthropometric, clinical and laboratory variables in study population.

*Parameters*	Whole patients (45)
Female/Male	14/31
Age, years (SD)	12.19 (3.05)
Weight, kg (SD)	68.13 (20.82)
BMI, Kg/m^2^ (SD)	28.33 (5.96)
WC, cm (SD)	86.59 (12.16)
AST, UI/L (SD)	35.45 (18.76)
ALT, UI/L (SD)	51.50 (43.92)
GGT, UI/L (SD)	22.71 (16.28)
Total Cholesterol, mg/dL (SD)	153.49 (35.02)
LDL Cholesterol, mg/dL (SD)	98.16 (31.75)
HDL cholesterol, mg/dL (SD)	43.15 (7.79)
Triglycerides, mg/dL (SD)	119.20 (62.41)
Glucose, mg/dL (SD)	83.07 (10.51)
Insulin (SD)	22.27 (12.78)

BMI = body mass index; WC = waist circumference; AST = aspartate aminotransferase; ALT = alanine aminotransferase; HDL = high-density lipoprotein cholesterol; LDL = low-density lipoprotein cholesterol.

We analyzed the expression of IGF-I and IGF-IR quantified as intensity *per* area of liver tissue by immunofluorescence in order to evaluate whether the expression of the two factors correlate with histologic features of NAFLD (i.e. steatosis, lobular inflammation, ballooning and fibrosis) and with NAS. The intra-hepatic expression of IGF-I and IGF-IR significantly correlated with fibrosis (Spearman’s r = 0.355, p = 0.024; Spearman’s r = 0.413, p = 0.0048).

### The expression of intra-hepatic IGF-I and IGF-IR increases in parallel with fibrosis progression

Obese children were subdivided according to the degree of fibrosis into three categories: stage 1 fibrosis (16 patients), stage 2 fibrosis (20 patients) and stage 3 fibrosis (9 patients). The main demographic, anthropometric and biochemical features of the study population at different stages of fibrosis are reported in Table 2.

**Table 2:** Anthropometric, clinical and laboratory variables in patients with different degrees of fibrosis.

*Parameters*	Fibrosis 1 (n = 16)	Fibrosis 2 (n = 20)	Fibrosis 3 (n = 9)	*P*
Female/Male	4/12	7/13	3/6	0.802
Age, years (SD)	12.19 (2.61)	12.53 (3.35)	11.44 (3.28)	0.639
Weight, kg (SD)	65.96(17.79)	67.09(18.30)	73.92(30.33)	0.646
BMI, SDS (SD)	1.84 (0.23)	2.01(0.78)	2.34(0.97)	0.199
BMI, Kg/m^2^ (SD)	26.51(4.14)	28.90(6.01)	30.15(8.03)	0.305
WC, cm (SD)	85.61(5.56)	86.06(10.14)	89.33(21.90)	0.770
AST, UI/L (SD)	29.33(13.01)	37.10(18.18)	42.00(26.19)	0.127
ALT, UI/L (SD)	41.13(36.40)	52.35(42.01)	66.89(58.21)	0.235
GGT, UI/L (SD)	17.19(7.98)	22.25(16.45)	33.56(22.42)	0.229
Total Cholesterol, mg/dL (SD)	157.50(33.74)	148.20(36.44)	158.11(36.47)	0.673
LDL Cholesterol, mg/dL (SD)	100.86(28.59)	87.80(36.42)	117.00(11.62)	0.092
HDL cholesterol, mg/dL (SD)	43.36(6.52)	43.33(8.70)	42.44(8.31)	0.956

BMI = body mass index; WC = waist circumference; AST = aspartate aminotransferase; ALT = alanine aminotransferase; HDL = high-density lipoprotein cholesterol; LDL = low-density lipoprotein cholesterol.

**Fig 1:**
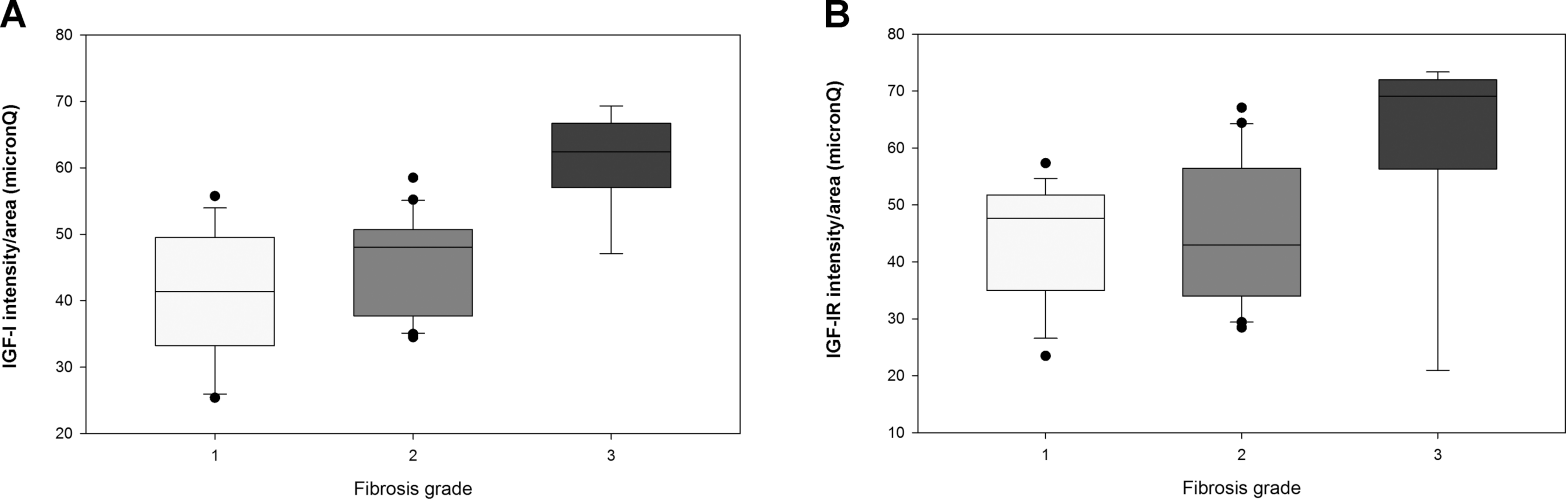
**IGF-I (A) and IGF-IR (B) intensity/area (micronQ) in the different stages of fibrosis.** Values are displayed as median and interquartile range (IQR).

As shown in Fig 1A and 1B and S1 Table and S1 Fig, the expression of IGF-I and IGF-IR quantified as intensity *per* area of liver tissue was significantly higher in children with stage 3 fibrosis compared to stages 1 and 2 (p < 0.001 and p = 0.007 respectively).

**Fig 2:**
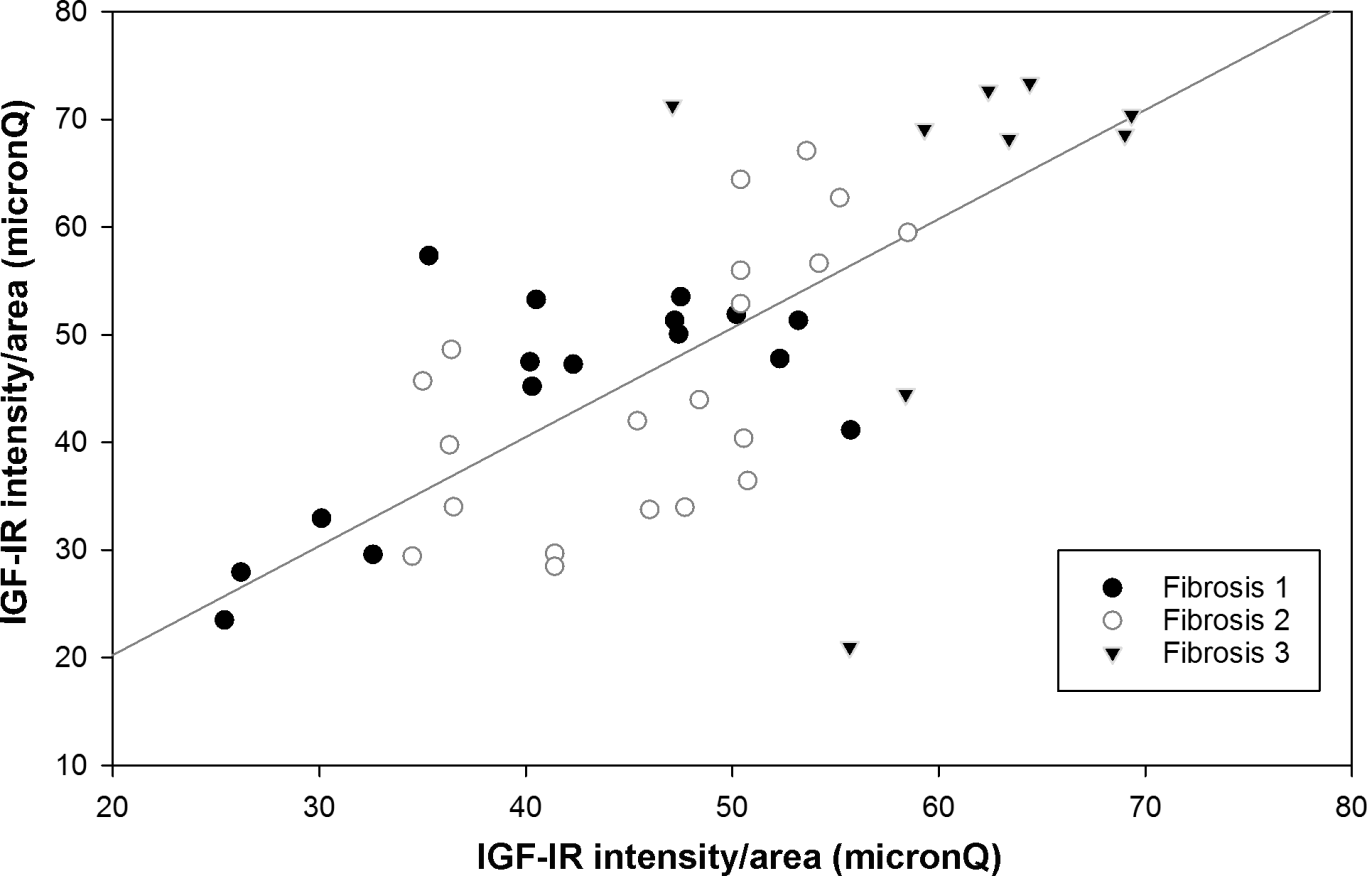
Correlation between IGF-I and IGF-IR expression, as intensity/area (micronQ) in the different stages of fibrosis.

Moreover, the increased expression of IGF-I correlated with the IGF-IR expression in all fibrosis stages (Spearman’s r = 0.61, p < 0.001) (Fig 2).

**Fig 3:**
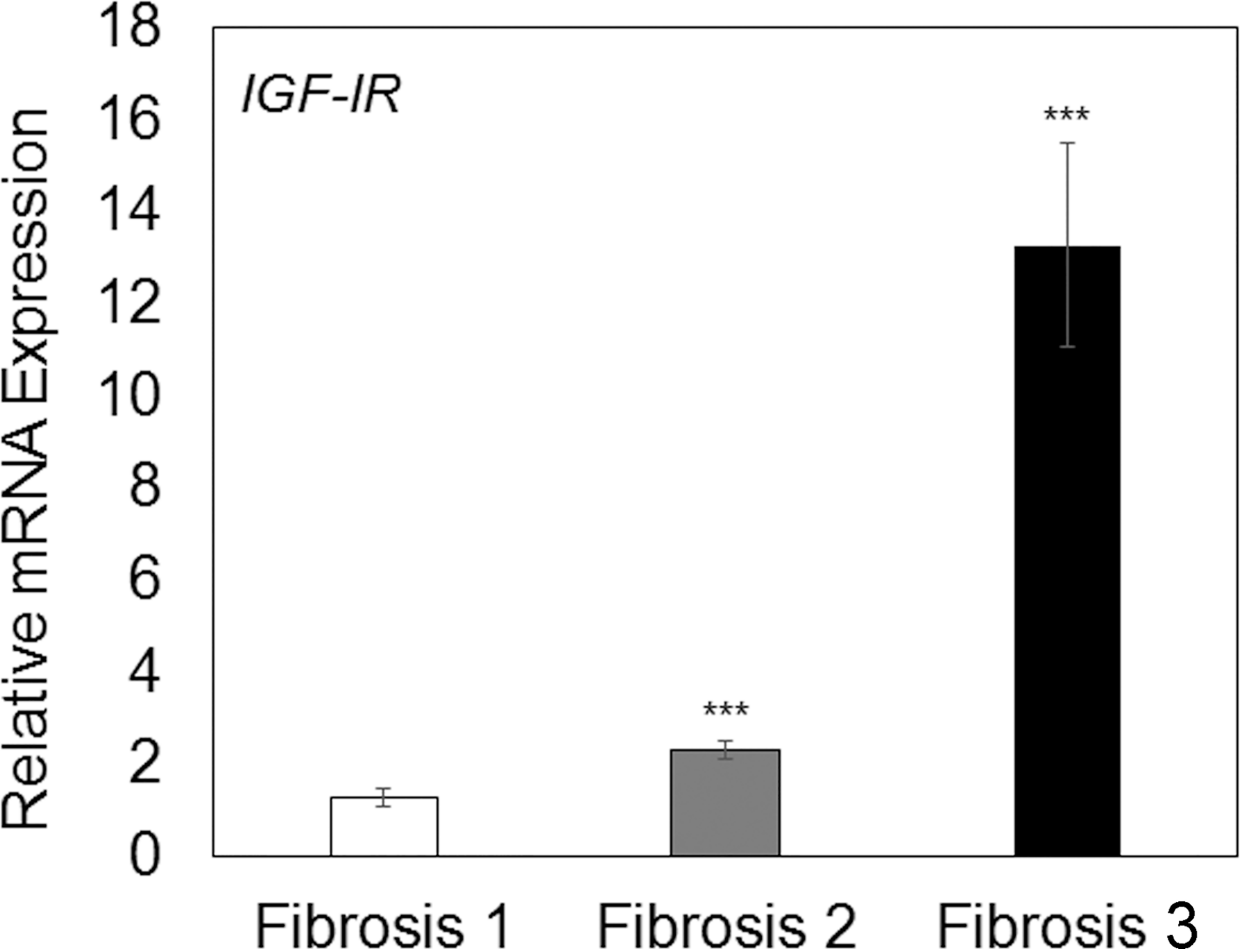
Relative mRNA expression of IGF-IR in the different stages of fibrosis. Data represent mean ± SD. ***P≤0.001 versus fibrosis; unpaired t-test.

A significant increase of expression *IGF-IR* mRNA was also found in advanced stage of fibrosis (2 and 3) compared to tissues with stage 1 of fibrosis (p < 0.001) (Fig 3).

### The increased expression of intra-hepatic IGF-I and IGF-IR is mainly associated to activated HSCs

**Fig 4:**
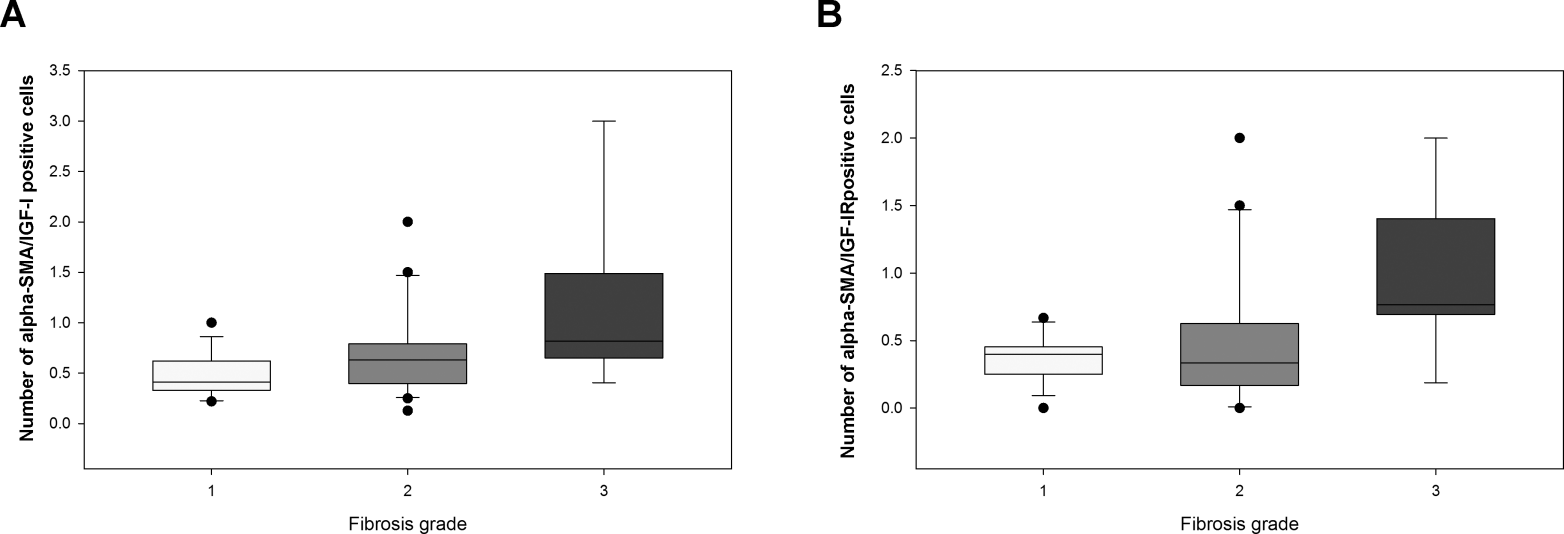
**Number of alpha-SMA positive cells (activated HSCs) expressing IGF-I (A) and IGF-IR (B) in the different stages of fibrosis.** Values are displayed as median and interquartile range (IQR).

Alpha-SMA expression is a reliable marker of HSCs activation in different type of fibrosis [37, 38]. As expected, the number of activated HSCs had an increasing trend with the progression of fibrosis (S2 Table). After normalization for the total number of HSCs, the number of HSCs expressing IGF-I was significantly increased in patients with stage 3 fibrosis compared to those with stage 1 fibrosis (p = 0.010) (Fig 4A and S2 Table and S2 Fig).

A significantly higher number of HSCs also expressed IGF-IR in stage 3 fibrosis compared to milder degrees (p = 0.008) (Fig 4B and S2 Table and S2 Fig).

## Discussion

Here, we have demonstrated for the first time in a pediatric cohort that liver IGF-I and IGF-IR expression correlates with fibrosis and increases in parallel with fibrosis progression, in the whole liver parenchyma and, more importantly, in activated HSCs, the main cell type involved in fibrogenesis. Fibrogenesis is a complex process of remodeling of liver architecture occurring during wound-healing response to either acute or chronic cellular injury [9]. Liver fibrosis is the result of an imbalance between liver repair and excessive extracellular matrix deposition mainly driven by HSCs [9]. HSC proliferation and their differentiation into collagen-producing myofibroblasts is an important event in the development and progression of liver fibrosis. Some *in vitro* studies have shown a proliferative and pro-fibrogenic potential of IGF-I on cultured cells, including quiescent HSCs [39]. However, other *in vitro* data suggest that IGF-I action on HSCs is dependent on their stage of differentiation [26]. On the other hand, the hepatoprotective and antifibrogenic potential of IGF-I has been shown by numerous studies. Sanz et al. [40] showed that targeted over-expression of IGF-I in activated HSCs of transgenic mice accelerates liver regeneration limiting fibrosis. Exogenous administration of IGF-I in experimental liver fibrosis improved liver function [21], reduced oxidative damage [21, 22], provided mitochondrial protection [23] and limited fibrosis [24, 25, 30] in rodent species, suggesting potential clinical applications of IGF-I.

Furthermore, in hepatocytes under normal conditions IGF-IR is present at low levels [41], but its expression is upregulated during pathological conditions, such as chronic hepatitis C and liver cirrhosis [42]. This up-regulation could be a mechanism of cytoprotection by which exogenous IGF-I could exert its well-described positive effects on the liver.

Evidence of a hepatoprotective effect of IGF-I derives also from human studies, showing that growth hormone replacement therapy reverts liver damage in GH deficient patients, for whom NAFLD is an important complication of the disease [18–20]. Moreover, IGF-I has a well-described function in lowering insulin resistance, that plays a pivotal role in the pathogenesis of NAFLD[43] and has been shown to increase albumin levels after 120 days of subcutaneous administration in cirrhotic patients [44].

The observation of increased expression of IGF-I in the liver in the present work is not in contrast with our previous data reporting that serum levels of IGF-I are inversely associated with the severity of NAFLD. Indeed, we found that IGF-I levels increase locally with the progression of fibrosis, suggesting a hepatocellular production/release of IGF-I, as a further attempt to induce a local repair of the damage. Further studies are required to investigate the real role of IGF-I/IGF-IR axis and mechanistic connection with other circulating factors and NAFLD-related damage.

Our work has a few potential limitations. First, we could not include a control group of healthy age-matched individuals in order to exclude the influence of unknown individual-related factors on IGF-I expression in the liver. Second, we did not assess the expression of factors that could confirm a hepatoprotective effect of IGF-I on liver cells and unravel the underlying mechanism, such as molecules involved in oxidative damage (i.e. inducible nitric oxide synthase and myeloperoxidase levels) or hepatocyte growth factor (HGF) that is stimulated by IGF-I [45] and limit fibrosis in vivo by stimulating hepatocyte regeneration [46]. These determinations as well as the assessment of circulating levels of IGF-I in this cohort of patients are perspectives that might build the bases for future studies.

In conclusion, our study is the first to analyze the local expression of IGF-I/IGF-IR in the liver of NAFLD children. We demonstrated for the first time that IGF-I and IGF-IR liver expression is up-regulated in pediatric NAFLD and particularly in HSCs. These findings give a new hint for the potential therapeutic use of IGF-I in pediatric NAFLD complicated by liver fibrosis.

## Supporting information

S1 Fig Representative image of IGF-I and IGF-IR expression in liver tissues from children with NAFLD. Magnification 20X.
(TIF)

S2 Fig Representative image of IGF-I/alpha-SMA and IGF-IR/ alpha-SMA co-expression in liver tissues from children with NAFLD. Magnification 20X.
(TIF)

S1 Table IGF-I and IGF-IR liver expression in patients with different degrees of fibrosis. (DOCX)

S2 Table Number of alpha-SMA positive cells and double IGF-I/alphaSMA and IGF-IR/alpha-SMA positive cells in patients with different degrees of fibrosis. (DOCX)

S1 Database Set of data by which results reported in the article can be reproduced. (XLSX)
