# Supplementary figures and images for: Expression of insulin-like growth factor I and its receptor in the liver of children with biopsy-proven NAFLD
Source: PLoS One. 2018 Jul 31;13(7):e0201566. doi: 10.1371/journal.pone.0201566 (PMC6067746; doi:10.1371/journal.pone.0201566)

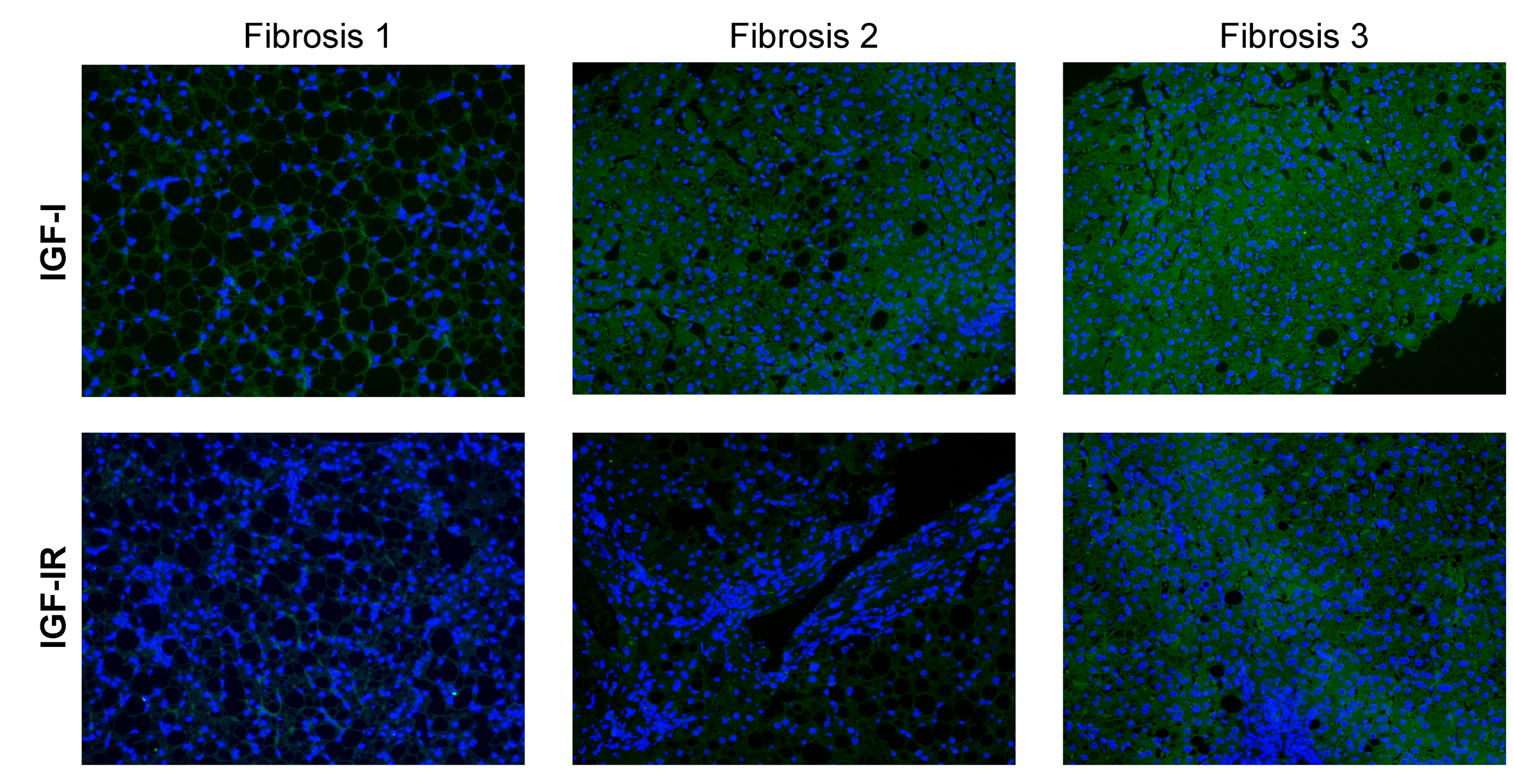

Supplement: S1 Fig — Magnification 20X. (TIF) [file pone.0201566.s001.tif]

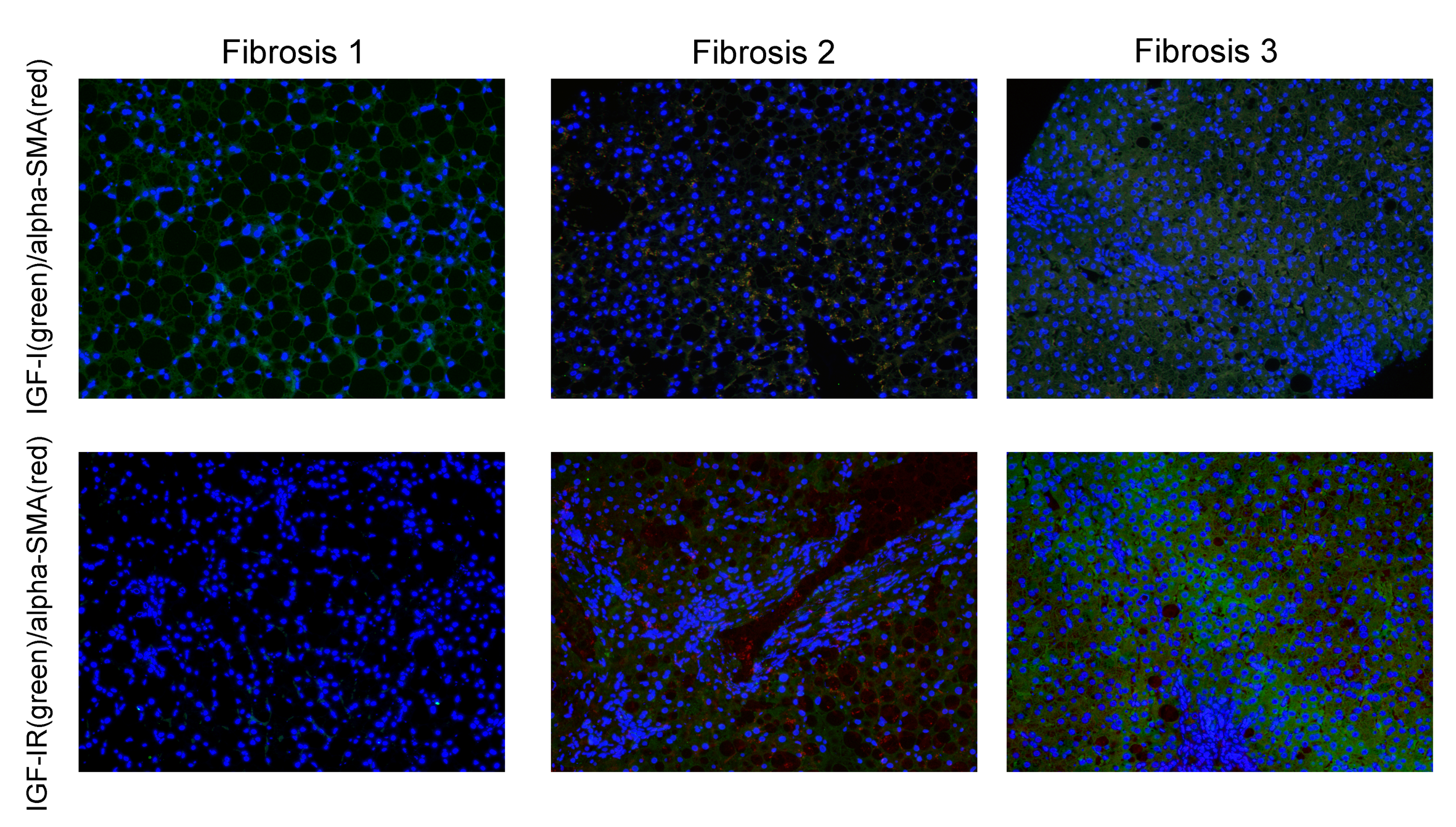

Supplement: S2 Fig — Magnification 20X. (TIF) [file pone.0201566.s002.tif]
