# Supplementary material for: Expression of insulin-like growth factor I and its receptor in the liver of children with biopsy-proven NAFLD
Source: PLoS One. 2018 Jul 31;13(7):e0201566. doi: 10.1371/journal.pone.0201566 (PMC6067746; doi:10.1371/journal.pone.0201566)
Supplement: S1 Table — (DOCX) [file pone.0201566.s003.docx]

**S1 Table. IGF-I and IGF-IR liver expression in patients with different degrees of fibrosis.**

|  | **Fibrosis 1**  **(n=16)** | **Fibrosis 2**  **(n=20)** | **Fibrosis 3**  **(n=9)** | ***P*** |
| --- | --- | --- | --- | --- |
| IGF-I intensity/area (micronQ) (SD) | 41.65(9.59) | 46.15(7.43) | 61.00(6.93) | **<0.001** |
| IGF-IR intensity/area (micronQ) (SD) | 44.47(10.37) | 45.26(12.50) | 62.10(17.75) | **0.004** |
