# Supplementary material for: Expression of insulin-like growth factor I and its receptor in the liver of children with biopsy-proven NAFLD
Source: PLoS One. 2018 Jul 31;13(7):e0201566. doi: 10.1371/journal.pone.0201566 (PMC6067746; doi:10.1371/journal.pone.0201566)
Supplement: S2 Table — (DOCX) [file pone.0201566.s004.docx]

**S2 Table. Number of alpha-SMA positive cells and double IGF-I/alphaSMA and IGF-IR/alpha-SMA positive cells in patients with different degrees of fibrosis.**

|  | **Fibrosis 1**  **(n=16)** | **Fibrosis 2**  **(n=20)** | **Fibrosis 3**  **(n=9)** | ***P*** |
| --- | --- | --- | --- | --- |
| alpha-SMA+ cells (SD) | 1.11(0.69) | 1.20(0.72) | 1.42(0.62) | 0.493 |
| IGF-I/alphaSMA+ cells (SD) | 0.47(0.25) | 0.68(0.36) | 1.26(0.31) | **<0.001** |
| IGF-IR/alphaSMA+ cells (SD) | 0.41(0.31) | 0.45(0.32) | 1.10(0.39) | **<0.001** |
| Ratio IGF-I/alphaSMA+ cells/ alpha-SMA+ cells (SD) | 0.48(0.23) | 0.70(0.45) | 1.13(0.81) | **0.010** |
| Ratio IGF-IR/alphaSMA+ cells/ alpha-SMA+ cells (SD) | 0.36(0.17) | 0.50(0.52) | 0.96(0.58) | **0.008** |
